# *Lactobacillus sakei* Pro-Bio65 Reduces TNF-α Expression and Upregulates GSH Content and Antioxidant Enzymatic Activities in Human Conjunctival Cells

**DOI:** 10.1167/tvst.10.6.8

**Published:** 2021-05-06

**Authors:** Roberto Iorio, Sabrina Petricca, Carla Luzi, Pierangelo Bellio, Loredana Cristiano, Claudio Festuccia, Gianfranco Amicosante, Giuseppe Celenza

**Affiliations:** 1 Department of Biotechnological and Applied Clinical Sciences, University of L'Aquila, L'Aquila, Italy; 2 Department of Life, Health, and Environmental Sciences, University of L'Aquila, L'Aquila, Italy

**Keywords:** *Lactobacillus sakei* (*L.SK*), human conjunctival epithelial cells, mitochondrial activity, reactive oxygen species (ROS), glutathione (GSH), enzymatic antioxidant defense, tumor necrosis factor alpha (TNF-α)

## Abstract

**Purpose:**

The study investigates the regulatory effects exhibited by lysate of *Lactobacillus sakei* pro-Bio65 (4%; *L.SK*) on the human conjunctival epithelial (HCE) cell line.

**Methods:**

Trypan blue and methylthiazol tetrazolium (MTT) methods were used to assess cell growth and viability. Mitochondrial membrane potential was assessed by JC-1 staining and cytofluorimetric detection methods. The antioxidant pattern and the intracellular reactive oxygen species (ROS) levels were analyzed by spectrophotometric and spectrofluorimetric methods. NF-κB luciferase activity was quantified by luminometric detection. NF-κB nuclear translocation, as well as mitochondrial morphology, were investigated by immunofluorescence using confocal microscopy. Cytokines and COX2 expression levels were determined by Western blot analyses.

**Results:**

This study demonstrates that *L.SK* exposure does not influence HCE cell proliferation and viability in vitro. *L.SK* paraprobiotic induces mild-low levels of intracellular ROS. It is coupled to changes in the mitochondrial membrane potential (ΔΨm), in a context of a regular mitochondrial-network organization. The negative modulation of tumor necrosis factor alpha (TNF-α) expression levels and rising antioxidant defense efficiency, mediated by the upregulation of glutathione (GSH) and increased antioxidant enzymatic activities, were observed.

**Conclusions:**

This study demonstrates that *L.SK* empowers the antioxidant endogenous efficiency of HCE cells, by the upregulation of the GSH content and the enzymatic antioxidant pattern, and concurrently reduces TNF-α protein expression.

**Translational Relevance:**

Although the obtained in vitro results should be confirmed by in vivo investigations, our data suggest the possibility of *L.SK* paraprobiotic application for promoting eye health, exploring its use as an endogen antioxidant system inducer in preventing and treating different oxidative stress-based, inflammatory, and age-related conditions.

## Introduction

The ocular surface (OS) is continuously exposed to the external environment and, therefore, to different microbial species. Although the ocular microbiome analysis is just getting started, culture-independent methods using 16S ribosomal RNA gene sequencing have shown the presence of a resident OS microbiota,^1^ which is, as in the gut, mouth, or skin, essential for eye health.

A protective immunoregulatory role for OS bacteria was recently suggested.^1^^,^^2^ Quantitative alterations of OS microbiota by environmental influences, host factors, antibiotics, and diseases may influence epithelial metabolism, proliferation, as well as survival. They may thus represent a cofactor in the pathogenesis of common ocular diseases.^3^^,^^4^ Epithelial cells act, de facto, as the coordinating server for the interplay between immune and bacteria cells.^5^

It is reasonable to hypothesize that probiotics or paraprobiotics may positively regulate ocular epithelial barrier function based on these complex interactions. They may represent a promising tool to prevent ocular disorders and improve eye functions and health by reducing pro-inflammatory cytokines, redox status impairment, and pathogenic bacterial overgrowth.^6^^,^^7^

Even though little is known, it has been suggested that the topical use of probiotics might regulate OS immune responses. For instance, a 4-week topical administration of *Lactobacillus acidophilus* eye drops ameliorate the clinical conditions in keratoconjunctivitis.^8^ The rationale behind the use of probiotics is based on their ability to produce bacteriocins, stimulate IgA production, and decrease pro-inflammatory cytokines by interacting with Toll-like receptors (TLRs).^9^

Scientific evidence demonstrates that probiotics have antioxidant properties,^10^^,^^11^ which positively influence epithelial cells by modulating the redox status. Commensal Lactobacilli induce physiological levels of reactive oxygen species (ROS) and consequent cell proliferation.^12^^–^^14^ Moreover, it has been demonstrated that Lactobacilli-induced ROS production leads to the activation of downstream cytoprotective signaling in gut epithelial cells.^15^^,^^16^ Such observations may be significant in considering the critical role of oxidative stress in inflammatory processes and, consequently, in the pathogenesis of ocular disorders, such as dry eye disease, cataracts, and pterygium, macular degeneration, keratoconus, diabetic retinopathy, and glaucoma.^17^^,^^18^

Although several health benefits are associated with probiotics, many concerns arose about shelf-life and safety problems.^19^ To reduce the possibility of risk of bacterial translocation and infection, the use of paraprobiotics, nonviable bacteria cells or bacterial components, is a valuable alternative. *Lactobacillus sakei* proBio-65 (*L.SK*) and its bioactive compound SEL001 exhibit anti-inflammatory effects in atopy and psoriasis.^20^^–^^25^ Antioxidant properties, as well as tyrosinase and α-glucosidase inhibitory effects of *L.SK*-derived exopolysaccharide, were also reported.^26^

In this study, the hypothesis that the bacteria lysate of *L.SK*, used at 4% as a protective ophthalmic solution (Immunodrop eye drops; FB Vision, Ascoli Piceno, Italy), could be able to stimulate the physiological generation of ROS and influence the enzymatic (superoxide dismutase, catalase, glutathione [GSH] peroxidase, GSH reductase, and GSH-S-transferase) and nonenzymatic (GSH levels) antioxidant defense mechanisms of human conjunctival epithelial cell line (Wong-Kilbourne derivative of Chang conjunctiva [ChWK]) was investigated. Considering the central role for mitochondrial ROS signaling in adaptive^27^ and innate immune responses^28^^,^^29^ and the immunomodulatory role of human conjunctival epithelial cells,^30^ the mitochondrial membrane potential (∆Ψ_m_) and morphology, as well as cytokines expression levels, were also examined.

## Materials and Methods

### Chemical Products and Antibodies

All reagents were purchased from Sigma-Aldrich (St. Louis, MO, USA) unless otherwise indicated.

Antibodies anti-COX2 were from Cell Signaling, Danvers, MA, USA; IL-1β and TNF-α from Santa Cruz Biotechnology, Inc., Dallas, TX, USA; IL-12A from Abcam, Cambridge, UK; IL-10, anti-rabbit or anti-mouse HRP-conjugated secondary antibody, Alexa Fluor 488 anti-rabbit IgG or 633 anti-rabbit IgG secondary antibodies from Immunological Sciences, distributed by Società Italiana Chimici, Rome, Italy, as well as ECL West Pico Plus.

### Cell Culture and Treatments

The Wong Kilbourne derivative (WKD) of the Chang Human Conjunctival Epithelial (HCE) cell line (ATCC CCL 20.2; clone 1-5c-4; WKD; ChWK) was supplied by European Collection of Authenticated Cell Cultures (ECACC 88021103). Lysate of *L.SK* was kindly provided by FB Vision (Ascoli-Piceno, Italy) and for experimental design, a final concentration of 4% (*L.SK*) in culture medium was used. HCE cells were cultured under standard conditions of 37°C, 5% CO_2_ humidified atmosphere. Cells were seeded at a density of 2 × 10^4^ cells/cm^2^ and maintained in the growth medium: Medium 199 supplemented with 2 mM glutamine and 10% fetal bovine serum (FBS), 1% penicillin (100 IU/mL) and streptomycin (100 µg/mL) until they reached a confluence close to 80%. Twenty-four hours after seeding, cells were treated with *L.SK*, for the times indicated below.

### Analyses of Cell Proliferation and Viability

HCE cells were seeded at a density of 2 × 10^4^/cm^2^ and cultures incubated in the presence or absence of *L.SK* extract to reach a final concentration of 4% in the culture medium. After 24 and 48 hours cell growth was assessed by counting and viability was determined by the trypan blue exclusion method, then confirmed by methylthiazol tetrazolium (MTT) assay, quantifying the metabolic efficiency of living cells.

### Detection of Intracellular Reactive Oxygen Species 

The ROS intracellular pool was detected using 2′,7′-dichlorofluorescein diacetate (DCFH_2_-DA) purchased from Molecular Probes (Eugene, OR, USA), as previously reported.^31^ After treatments (30 minutes and 2 hours), cells were incubated with 1 µM DCFH_2_-DA, at 37°C for 30 minutes. After collecting cells, samples (2 × 10^5^ cells) were washed twice in ice-cold PBS and sequentially analyzed by flow cytometry. The fluorescence intensity was acquired with a Perkin-Elmer LS-50B spectrofluorometer, setting the excitation and emission wavelengths at 502 and 524 nm, respectively. Cells treated with 200 µM tert-butyl hydroperoxide (t-BHP) for 1.5 hours were used as the positive control.

### Enzymatic Assays

After treatments with *L.SK* (2 hours of incubation), cells were washed with cold phosphate-buffered saline (PBS), resuspended in 50 mM Tris–HCl, pH 7.4, 1 mM EDTA, 1% (v/v) Triton X-100 and then disrupted by 3 consecutive cycles of freeze-thawing. After centrifugation at 17,000 g for 10 minutes at 4°C, the supernatant was collected for protein determination and subsequently analyzed.

The GSH reductase activity of cell extracts was assayed spectrophotometrically by following nicotinamide adenine dinucleotide phosphate (NADPH) oxidation at 340 nm at 25°C.^32^ The assay mixture consisted of 50 mM potassium phosphate buffer (pH 7.0), 1 mM EDTA, 1 mM glutathione disulfide (GSSG), 0.2 mM NADPH, and the appropriate amount of the extracted proteins.

Total (Se-dependent and Se-independent) GSH peroxidase activity was evaluated spectrophotometrically by measuring the NADPH oxidation at 340 nm. The reaction mixture contained 50 mM potassium phosphate buffer (pH 7.0), 1 mM EDTA, 0.2 mM NADPH, 1 mM GSH, 0.01 U/mL GSH reductase, 70 µM t-BHP, and the appropriate amount of the extracted proteins.^33^ The glutathione transferase activity was assayed by measuring GSH conjugation rate to 1-chloro-2,4- dinitrobenzene at 340 nm. The reaction mixture contained 0.1 M potassium phosphate buffer, pH 6.5, 2 mM GSH, 1 mM 1-chloro-2,4-dinitrobenzene, and aliquots of supernatant.^34^

The catalase activity was assayed by monitoring the decomposition of 10 mM at 240 nm, as described by Aebi.^35^ One unit was defined as 1 µmol of H_2_O_2_ reduced/min at 25°C.

The Superoxide Dismutase activity was assayed by a colorimetric activity assay (ThermoFisher Scientific - Life Technologies Corp., Carlsbad, CA, USA) as indicated by the manufacturer. The colored product was read at 450 nm. The enzymatic activities were expressed as nmol/min/mg protein (CAT, GR, GPx, and GST) or U/mg protein (superoxide dismutase [SOD]).

### Detection of GSH and GSSG Intracellular Content

The GSH and GSSG intracellular content were detected using a glutathione colorimetric detection kit (ThermoFisher Scientific - Life Technologies Corp.), as indicated by the manufacturer. The experiments were conducted as the manufacturer instructions at 30 minutes and 2 hours of incubation with *L.SK*.

### Western Blot Analysis

Total proteins were extracted from HSE cells after 6 hours incubation with *L.SK* by a lysis buffer containing 10 mM Hepes pH 7.2, 142 mM KCl, 1 mM EDTA, 5 mM MgCl2, 1 mM EDTA, 1 mM PMSF, and a suitable cocktail of protease inhibitor. The extracts were run on a 12% SDS-PAGE and transferred onto PVDF membrane and at RT for 1 hour with 5% nonfat dry milk in TBST containing 0.1% Tween-20. After washing, filters were incubated O/N at 4°C with primary antibodies anti-COX2 diluted 1:1000, IL-12A diluted 1:1000, IL-1β and TNF-α diluted 1:1000 and 1:500, respectively, IL-10 diluted 1:5000, in 5% nonfat milk in TBST 0.1% Tween-20. Membranes were then washed and incubated for 1 hour at room temperature (RT) with the corresponding anti-rabbit (1:5000) or anti-mouse (1:10000) HRP-conjugated secondary antibody. ECL West Pico Plus chemiluminescent substrate was used to detected signal with a ChemiDoc XRSplus imaging system (Bio-Rad Laboratories, Hercules, CA, USA). The blot bands were quantified by ImageJ software (US National Institutes of Health, Bethesda, MD, USA) and normalized with the ß-actin as a loading control.

### Flow Cytometry Assessment of ΔΨ_m_

Changes in the ΔΨ_m_, in samples incubated for 2 hours with *L.SK*, were analyzed using the JC-1 lipophilic cation dye, as previously reported.^36^ Samples (1 × 10^6^ cells) were incubated with 3 µM JC-1 (Molecular Probes, Eugene, OR, USA), for 30 minutes at 37°C in a humidified atmosphere, collected, washed in PBS, and analyzed by flow cytometry. The fluorescence signals of JC-1 monomers and aggregates were detected through the FL-1 (525 ± 5 nm bandpass filter) and FL-2 channels (575 ± 5 nm bandpass filter). Carbonyl cyanide 3-chlorophenylhydrazone (CCCP) was used at a final concentration of 8 µM for 1 hour, at 37°C to provide a positive control for the abolishment of the ΔΨ_m_. All experiments were performed with a FACS Calibur instrument (Becton Dickinson Instruments Inc.), and data for ΔΨ_m_ assessment were acquired by Cell Quest software program (Becton Dickinson Instruments Inc.). A minimum of 1 × 10^4^ cells was analyzed, by forward and side scatter channels, gated on the significant population of normal size cells.

### Dual-Luciferase Reporter Assay for Detection of NF-κB Activity

Transient transfection followed by luciferase assay was performed to determine the NF-κB activity rate. Cells were seeded in 96-wells microplate and incubated O/N to reach greater than 50% confluency. Co-transfection of the plasmids NF-κB-RE Firefly luciferase reporter vector and *Renilla* as control vector was performed using FuGENE HD transfection reagent (Promega, Madison, WI, USA) accordingly to the manufacturer's instructions. Cells were incubated at 37°C and allowed to recover for 24 hours before administering treatments (2 hours of incubation with *L.SK* extract*)*. The expression of luciferase enzyme activated by NF-κB binding was determined using the Dual-Luciferase Reporter Assay System (Promega), by following the manufacturer's protocol. The results of NF-κB activity were expressed as the relative promoter-luciferase activity, normalizing for *Renilla* activity.

### Confocal Fluorescence Microscopy

Cells seeded on coverslips, after O/N incubation were treated with *L.SK* for 2 hours. As previously reported,^37^ control and treated cells were fixed with 4% paraformaldehyde in PBS for 10 minutes at RT, permeabilized with 0.1% Triton X-100 in PBS, for 5 minutes at RT, and the nonspecific binding sites were blocked with 10% bovine serum albumin (BSA) in PBS, for 10 minutes at RT. After washings, cells were incubated with, rabbit anti-TOMM20 (ThermoFisher Scientific, Rockford, IL, USA), or with rabbit anti-NF-κB (Cell Signaling, Danvers, MA, USA) primary antibodies, diluted 1:500 in blocking solution, O/N at 4°C. After washings primary antibodies were revealed by Alexa Fluor 488 anti-rabbit IgG or 633 anti-rabbit IgG secondary antibodies, respectively (Immunological Sciences, distributed by Società Italiana Chimici, Rome, Italy), diluted 1:2000 in blocking solution, for 30 minutes at RT. Controls were performed by omitting the primary antibody. Coverslips were mounted with Vectashield Mounting Medium containing DAPI (Vector Laboratories, Burlingame, CA, USA) and observed at a Leica TCS SP5 confocal microscope (Leica, Mannheim, Germany). Data for mitochondrial morphology assessment were acquired by Leica LAS AF software and a minimum of 20 images for each determination were analyzed.

### Statistical Analyses

All experimental analyses were performed at least in three independent determinations. Unless otherwise indicated, data reported in this study are expressed as the mean ± standard error (SE). To test the statistical significance of differences between group means, the Sigma Stat 2.03 (SPSS, Chicago, IL, USA) was used. The comparison between control and treated groups was performed by Student's *t*-test, whereas comparisons between multiple groups were performed by the ANOVA test, followed by Dunnett's Method. ANOVA on ranks (Kruskal-Wallis test) followed by Dunnett's test was used to analyze GSH/GSSG ratio. Mann-Whitney Rank Sum Test was used for ΔΨ_m_ assessment (mean fluorescence intensity [MFI]). Any *P* < 0.05 was considered as a value that was statistically significant.

## Results

### Proliferation and Viability of HCE Cells are not Affected by the Presence of *L.SK*

**Figure 1.**
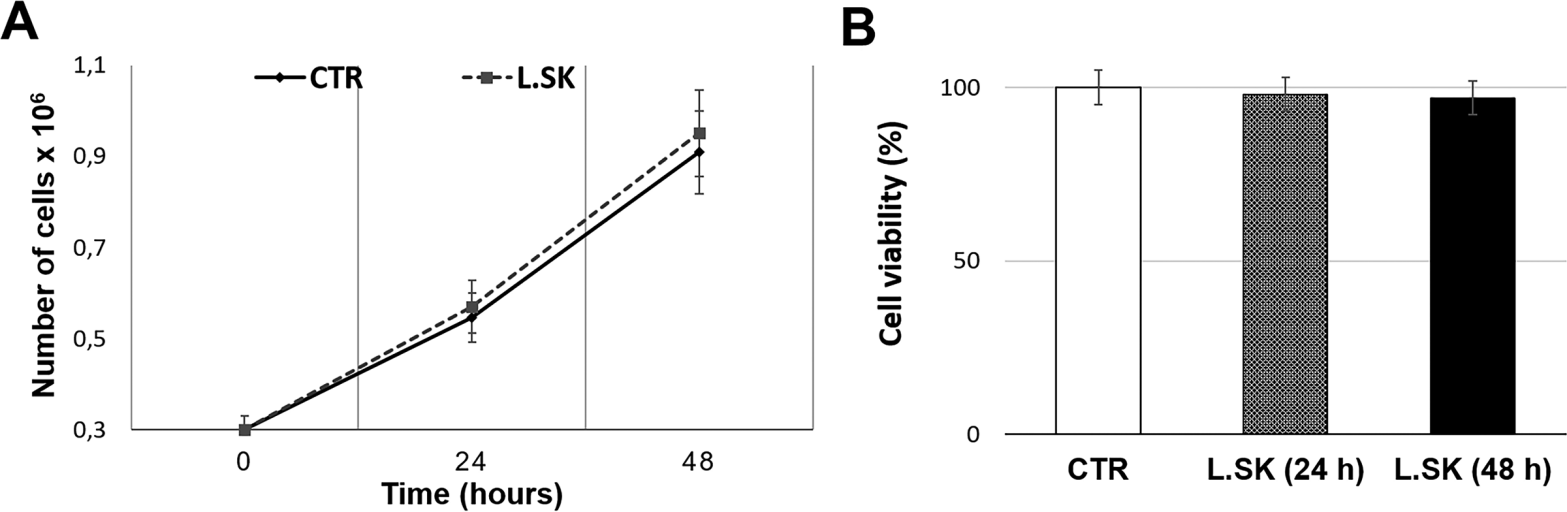
***L.SK* treatment does not induce changes in HCE cell proliferation and viability.** Cell growth (**A**) and viability (**B**) rate, in control (CTR) and *L.SK*-exposed HCE cells incubated for 24 and 48 hours. Results are the media of three independent experiments ± SE. Student's *t*-test.

HCE cells were exposed for 24 hours and 48 hours with *L.SK* and cell growth and viability were evaluated by trypan blue exclusion test and MTT assay, respectively. As shown in Figures 1A and 1B, no significant differences were found when comparing exposed groups with control groups.

### *L.SK* Induces Mild-Low Levels of Intracellular ROS in HCE Cells

**Figure 2.**
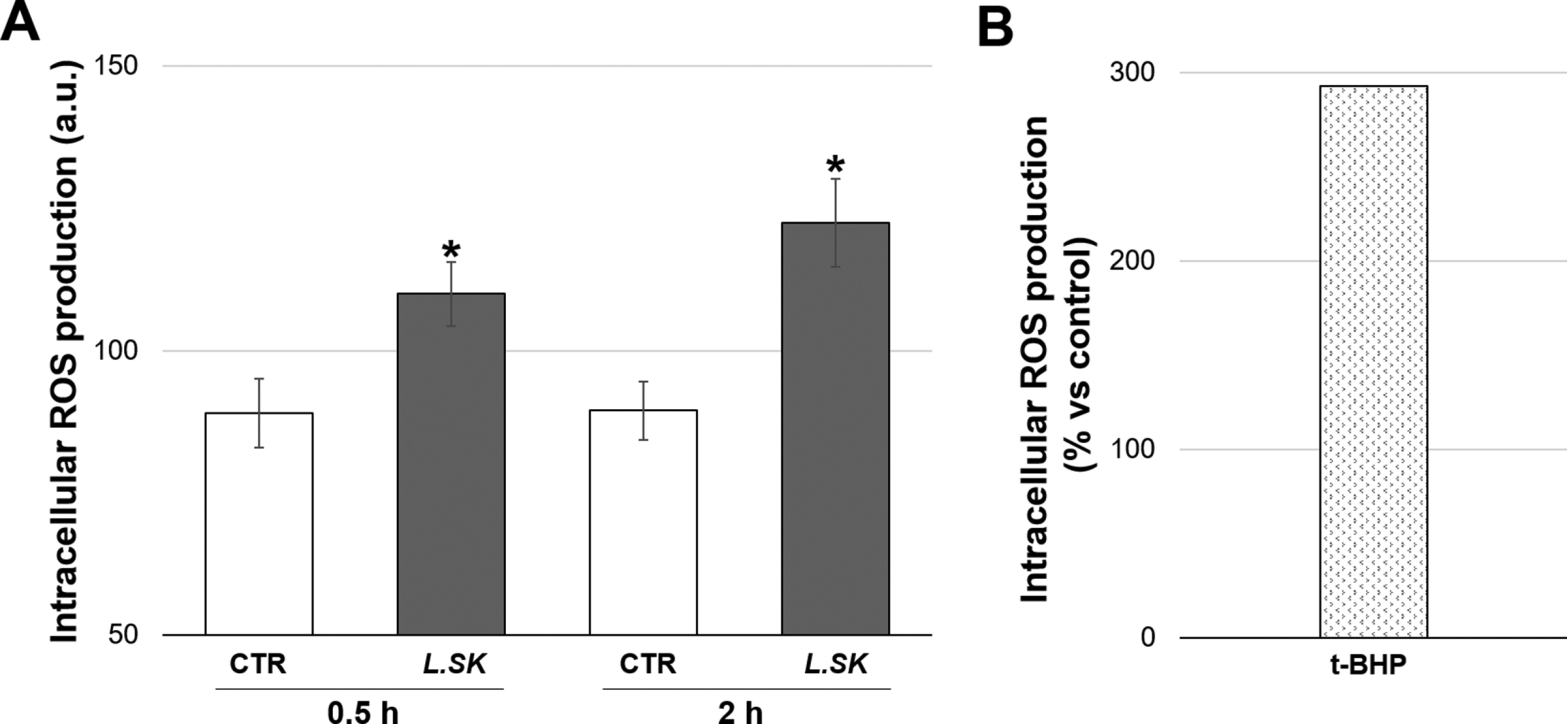
***L.SK* treatment induces mild-low levels of ROS in HCE cells.** (**A**) ROS production in HCE cells incubated in the absence (control [CTR]) or in the presence of *L.SK* for 0.5 and 2 hours; intracellular ROS were measured by fluorimeter, detecting DCFH_2_-DA fluorescence, and determined in terms of fluorescence intensity (arbitrary units [a.u.]). Values from three independent experiments are expressed as the media ± SE; Student's *t*-test; *P < 0.05 (**B**) Cells were incubated 1.5 hours with 200 µM t-BHP used as positive control for the ROS generation, expressed as percentage versus control.

Recent reports highlighted the ability of *Lactobacillus* probiotic to influence ROS production in host cells, causing changes in epithelial cells redox status. HCE cells were thus incubated in the presence of *L.SK* for 30 minutes and 2 hours and subsequently analyzed for DCFH_2_-DA fluorescence. As indicated in Figure 2, *L.SK* induced a slight but significant increase of intracellular ROS generation, already visible after 30 minutes of incubation (30 minutes approximately 24%; 2 hours approximately 30%). Samples treated for 1 hour with 200 µM t-BHP were used as a positive control for ROS production.

### *L.SK* Induces a Decrease of Mitochondrial Membrane Potential (∆Ψ _m_) in a Context of a Regular Mitochondrial Network Organization

**Figure 3.**
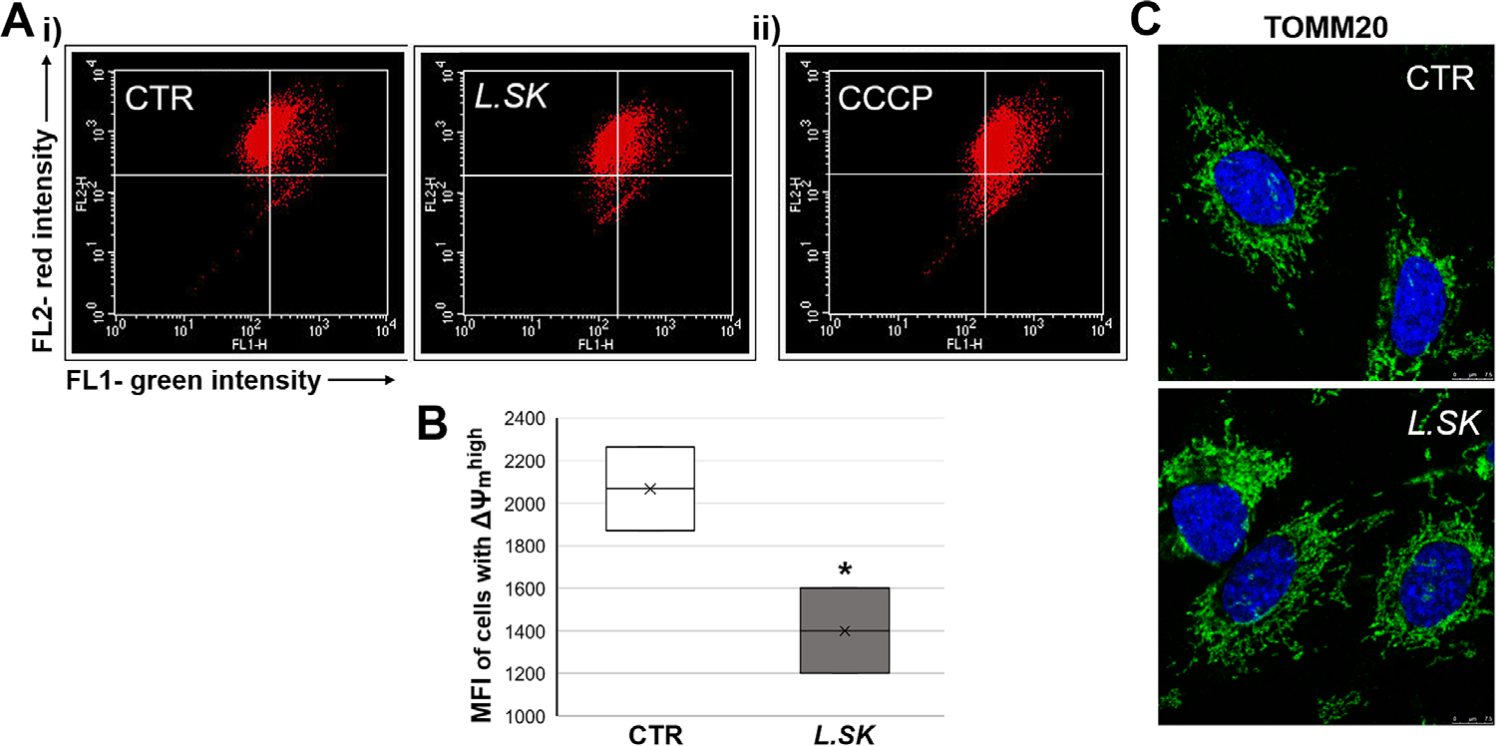
**Differences in the mitochondrial membrane potential (ΔΨ_m_) induced by *L.SK* treatment do not reflect alterations of morphology**. In panel (**A**), representative images of (i) not treated (control [CTR]) and treated cells with *L.SK* for 2 hours, or with (ii) 8 µM CCCP for 1.5 hours used as positive control for the dissipation of ΔΨ_m_. (**B**) A significant decrease of the mean fluorescence intensity (MFI) was revealed in populations with high ΔΨ_m_ of treated cells. Data from three independent experiments are expressed as the media ± SE. Mann-Whitney Rank Sum Test; *P < 0.05. In panel (**C**), immunofluorescence staining with the mitochondrial marker, TOMM20 (*green*) in control and treated (2 hours) HCE cells, examined at confocal microscopy. Nuclei (*blue*) were stained with DAPI.

To evaluate whether the *L.SK*-induced ROS were correlated with modifications in ∆Ψ_m_, the mitochondrial function was detected by JC-1 staining. As shown in Figure 3B, the MFI of cells with high ∆Ψ_m_ (∆Ψ_m_^high^_)_ were significantly lower (approximately 34%) in *L.SK*-treated cells (2 hours) when compared with the control groups. Figure 3A(i) shows that *L.SK* substantially decreased the MFI of the cell with ∆Ψ_m_^high^. Figure 3A(ii) reveals CCCP-treated cells used as a positive control for the dissipation of ∆Ψ_m_.

Decrease of ∆Ψ_m_ suggests changes in mitochondrial dynamics.^37^^,^^38^ Confocal analyses of immunofluorescent labeled mitochondria with the anti-TOMM20 antibody did not show significant differences between control and experimental groups (Fig. 3C). Compared with the control groups, the tubular morphology for experimental groups suggests mitochondrial homeostasis maintenance.

### *L.SK* Treatment Does not Result in NF-κB Pathway Involvement and Leads to a Negative Modulation of TNF-α Expression Levels

Given the existence of a feedback-loop expressed by ROS/NF-κB signaling, and the possible relationship between mitochondria and innate immunity, we have turned our analyses on monitoring an NF-κB possible activation (2 hours) in parallel to a cytokine investigation (6 hours).

**Figure 4.**
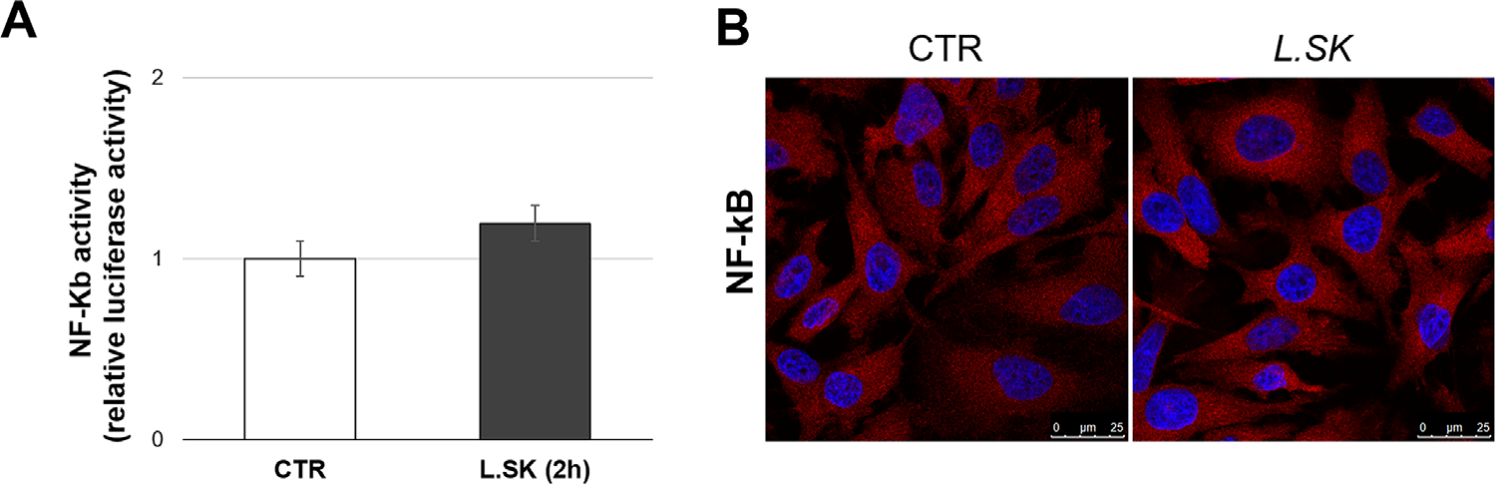
***L.SK* treatment in HCEC does not result in an involvement of NF-κB pathway.** (**A**) The NF-κB luciferase activity, of not treated (control [CTR]) and treated cells with *L.SK* for 2 hours of incubation. Results are the media of three independent experiments ± SE; Student's *t*-test; (**B**) representative images, at confocal microscopy, of control and treated (2 hours) HCE cells stained with the anti-NF-κB antibody (*red*) and DAPI for the nuclei (*blue*).

**Figure 5.**
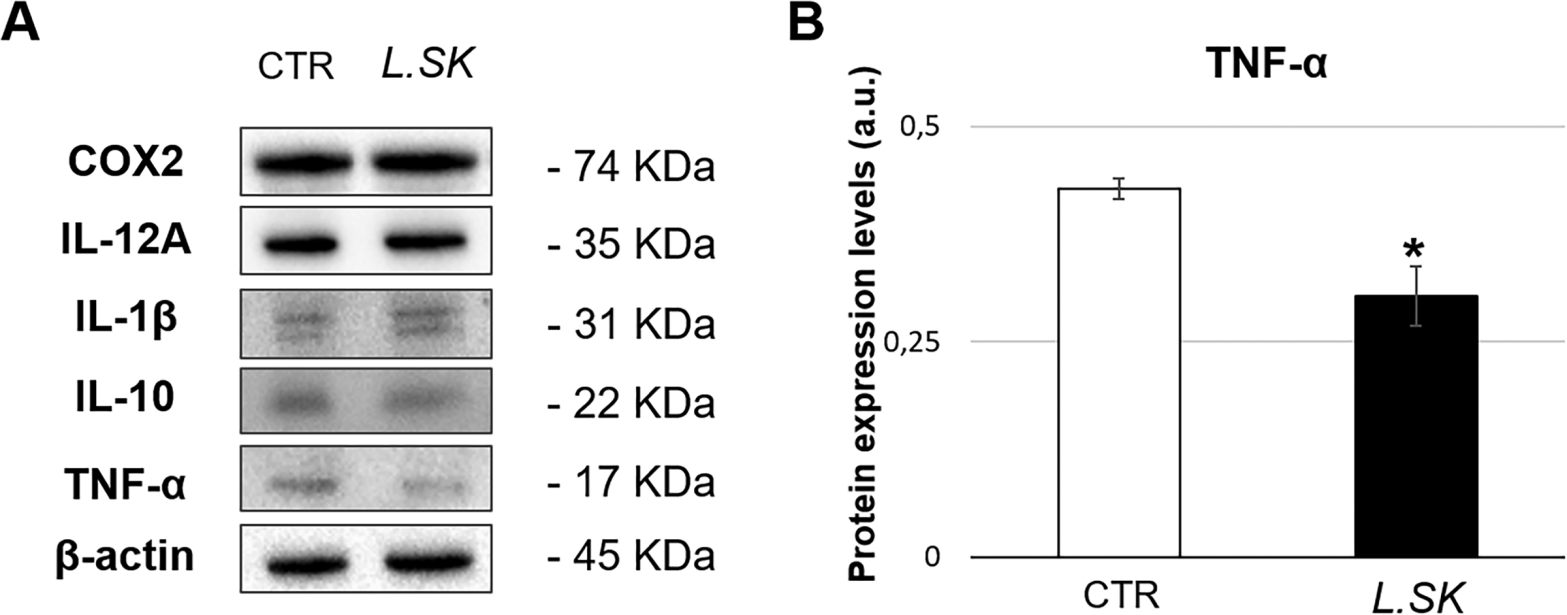
***L.SK* treatment down** **regulates TNF-α protein expression levels in HCE cells.** (**A**) Representative images of COX2 and cytokines blot band in not treated (control [CTR]) and treated samples with *L.SK* for 6 hours of incubation. (**B**) Protein expression levels of TNF-α in *L.SK* treated cells at 6 hours of incubation, compared with the controls (arbitrary units [a.u.]). Values from three independent experiments are expressed as the media ± SE; Student's *t*-test; *P < 0.05.

Our results of the NF-κB activity, in terms of both DNA-binding affinity as luciferase induction (Fig. 4A) and nuclear translocation (Fig. 4B), failed to reveal its direct involvement. Accordingly, Western blot analyses of the protein expression levels of pro- and anti-inflammatory monitored cytokines (IL-1β, IL-12A, TNF-α, and IL-10) and COX2 did not exhibit substantial variations, except for TNF-α; in particular, as shown in Figure 5B, the TNF-α expression levels, at 6 hours of incubation, were significantly reduced in treated samples when compared with the controls.

### *L.SK* Exposure Upregulates the Reservoir of GSH Content and Reinforces the Enzymatic Antioxidant Defense Efficiency in HCE Cells

**Figure 6.**
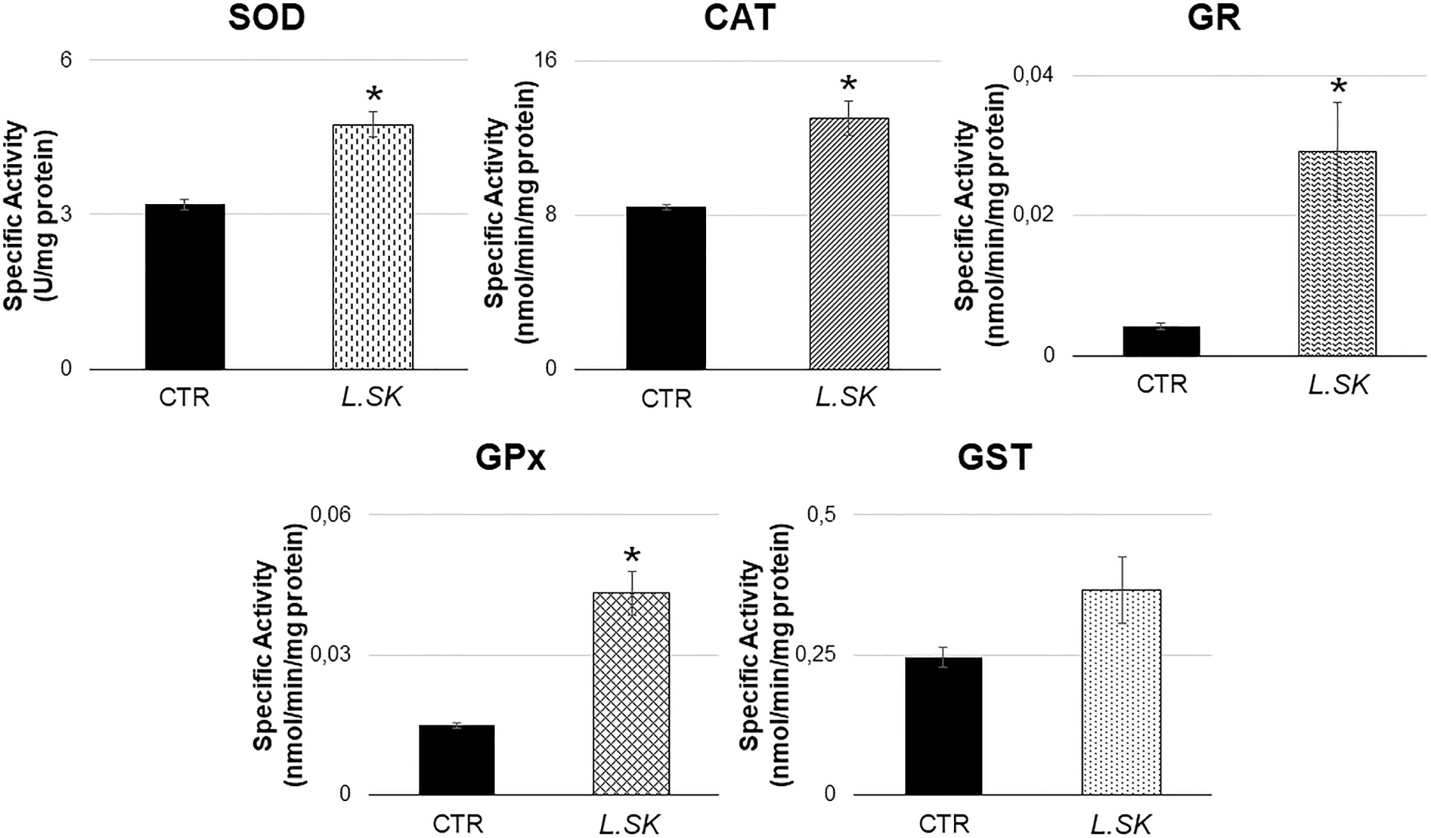
**Enzymatic activity of the antioxidant pattern is up-regulated by *L.SK* in HCE cells.** Cells were incubated in the absence (control [CTR]) or in the presence of *L.SK* and after 2 hours of treatment the enzymatic activities were assayed by spectrophotometric-based methods. Data are expressed as nmol/min/mg protein or U/mg protein, as indicated. Values from three independent experiments represent the media ± SE; Student's *t*-test; *P < 0.05.

To further characterize the *L.SK-*induced changes in HCE redox status, enzymatic and not enzymatic assays of the antioxidant system were performed. Generally, a positive effect was already observed at 30 minutes of incubation (data not shown) for enzymatic assay, up to 2 hours. In particular, as described in Figure 6, CAT, SOD, GR, and GPx enzymatic activities increased significantly in *L.SK*-exposed samples (2 hours) when compared with control groups. GST activity shows the same behavior, even though experimental values were not significant. Overall, these results suggest an improved efficacy of the enzymatic antioxidant system. Besides the strong increase in the single specific activities, we also observed a constant CAT/SOD ratio (control [CTR] = 2.657 ± 0.078; *L.SK* = 2.757 ± 0.197), which suggests the conservation of the redox homeostasis. Furthermore, the raise in GPx/SOD ratio (CTR = 0.00467 ± 0.0000667; *L.SK* = 0.0332 ± 0.0234), although not significant, is indicative of a higher potential in scavenging defense.

**Figure 7.**
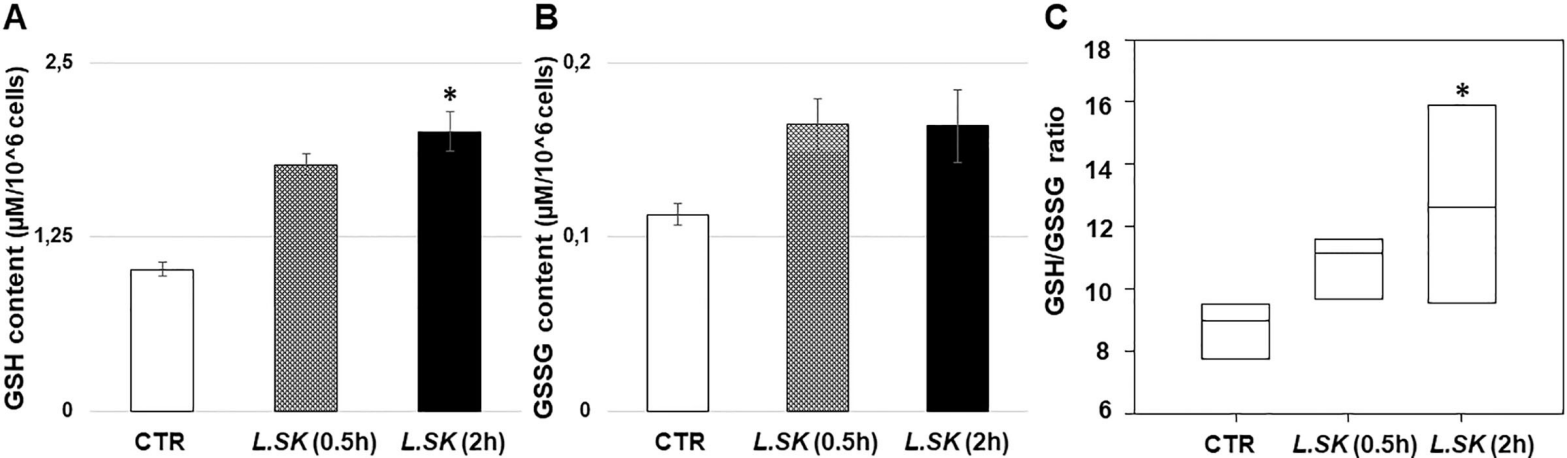
**Stimulation by *L.SK* noticeably increases GSH content levels with a further significative gain in GSH/GSSG ratio.** Levels of (**A**) reduced (GSH), (**B**) oxidized (GSSG) glutathione, and (**C**) GSH/GSSG ratio in control and *L.SK-*treated groups after 30 minutes and 2 hours of incubation. Values from three independent experiments are expressed as the media ± SE; **A** and **B** ANOVA test, followed by Dunnett's Method; (**C**) ANOVA on ranks (Kruskal-Wallis test) followed by Dunnett's test; *P < 0.05.

*L.SK* treatment (2 hours) induced positive and significant changes in reduced glutathione content, without modifying the oxidized form levels (Figs. 7A, 7B). In line with this, a positive shift in the cellular GSH/GSSG redox balance was recorded (Fig. 7C), also confirming the absence of an oxidative stress condition. This statement was supported by an average level of lipid peroxidation observed in *L.SK* experimental groups (data not shown).

## Discussion

Redox networks constitute an adaptive system to respond to the environment. It is not by chance that ROS are generated and dynamically modulated in response to bacteria. This study shows how the lysate of *L.SK* elevates low intracellular ROS levels in human conjunctival epithelial cells without affecting cell growth and viability. More important, no changes in GSSG levels and no lipid peroxidation phenomena have also occurred. This result ties well with previous reports, showing that probiotics may influence cellular homeostasis by increasing ROS production.^12^^–^^14^ It is also known that Lactobacilli-induced ROS stimulate epithelial cytoprotection^15^ influencing epithelial development.^16^^,^^39^^,^^40^

Mitochondrial oxidative metabolism is the primary source of cellular ROS. Several reports highlight the central role of mitochondrial ROS (mROS)-dependent signaling in various biological systems.^41^^,^^42^ Mitochondria can buffer cellular H_2_O_2_ by using GSH and catalase system acting as ROS stabilizing devices due to their ability to quench extramitochondrial redox signals originating from physiological stimuli.^43^ Innate immune responses are also actively regulated by mitochondria, which act as central hubs.^28^^,^^29^ For example, the activation of TLRs, responsible for recognizing microbe-associated molecular patterns (MAMPs), generates mitochondrial depolarization and mROS production.^29^ Conjunctival epithelial cells express multiple TLRs^44^ and Lactobacilli or their cellular components, can elicit immunostimulatory effects in macrophages stimulating TLR2 signaling.^45^^–^^47^ In line with this, the *L.SK*-induced ROS occurs in parallel with a significant decrease in ∆Ψ_m_ without affecting mitochondrial morphology, also confirming the preservation of mitochondrial functions. *L.SK* downregulates the productions of TNF-α and does not stimulate the expression of pro-inflammatory cytokines IL-1ß and IL-12A. These results are in line with Kim and colleagues who reported a significant decrease of TNF-α production by live or heat-killed *L.SK* in mast cells and animal models.^21^ These findings should be examined in detail considering the dominant role of epithelial cells in allergic diseases, exerted via cytokines and adhesion/effector molecule release.^48^

Given the importance of NF-κB in TLR signaling pathways, we also investigated its nuclear translocation and activity. No significant modulation of NF-κB activity was found, suggesting that other mechanism may be involved in *L.SK*-induced effects. On the other hand, ROS can simultaneously stimulate and extinguish NF-κB signaling. We should consider that *L.SK* may affect nuclear factor-erythroid 2-related factor-2 (Nrf2) pathway linked to ROS.^49^ The activation of Nrf2 may be the reason for the *L.SK*-mediated cellular antioxidant capacity modulation.

Protective and anti-oxidative effects of lactic acid bacteria have also been suggested.^50^^,^^51^ In particular, *Lactobacillus plantarum* FC225 increased SOD and glutathione peroxidase (GPx) activity in mice.^52^

For instance, we observed that exposure of conjunctival epithelial cells to *L.SK* significantly enhances enzymatic antioxidant system activity in terms of SOD, CAT, GR. and GPx. In accord with the notion of “hormesis,” upon exposure to *L.SK*-induced mild stress, epithelial cells would activate a protective response increasing cellular antioxidant capacity. This hypothesis is supported by the maintenance and the increase of CAT/SOD and GPx/SOD indexes. SOD, GPx, and CAT are the primary antioxidant enzymes directly involved in ROS detoxification and redox maintaining homeostasis, protecting conjunctival cells from cytotoxicity. It is known that the deregulation of this system leads to lipid peroxidation membrane damage and the development of OS diseases, such as dry eye syndrome and allergic conjunctivitis.^53^

Intracellular GSH content and GSH/GSSG ratio were also found incremented, preliminarily indicating the absence of oxidative stress. The GSH is essential to preserve the redox homeostasis and cellular mechanisms, such as redox signaling pathways, cell proliferation and apoptosis, xenobiotics detoxification, protein folding, and immune response.^54^ The central role of GSH in protecting OS from injuries induced by oxidative stress is recognized.^55^^,^^56^ GSH is mainly secreted by conjunctival cells, representing the most abundant endogenous antioxidant in the tear film. The involvement of GSH in the pathogenesis of pterygium^57^ and keratoconus^58^ has been reported. For instance, increased oxidative stress, lipid peroxidation, and decreased GSH are associated with keratoconus.^59^ The importance of GSH in the lens homeostasis is well known, and the repercussion of its depletion on the ability of the crystalline uptake of reduced GSH from the neighboring tissues has also been related to aging.^60^^–^^63^ The cellular balance between GSH and its disulfide form, GSSG, is an indicator of oxidative stress^64^ depending on GSH depletion, GSSG/GSH recycling, and synthesis of GSH. GSH acts as cofactor of antioxidant enzymes and the *L.SK*-induced increased activity of GPx and GST would have led to enhanced depletion of GSH in favor of GSSG production. In this situation, however, the increase in GR activity would compensate for GSH consumption due to its high efficiency in recycling GSH from GSSG.

In summary, we demonstrated that *L.SK* elevates human conjunctival epithelial cell antioxidant capacity, as manifested by the upregulation of the GSH content and the enzymatic antioxidant system. Moreover, it reduces TNF-α protein expression and pro-inflammatory cytokine. Although further studies are needed to investigate the molecular mechanism underlying *L.SK*-induced effects, possible roles of mitochondrial activity and Nrf2 signaling pathway are hypothesized.

Finally, although the obtained in vitro results should be confirmed by in vivo investigations, our data emphasize the possibility to use paraprobiotics for promoting eye health. This path should be explored in light of the close relationship between oxidative stress and many ocular alterations, including cataracts, keratoconus, retinopathy, age-related degenerations, and dry eye disease.

### Therapeutic Potential

Aging is characterized by progressive ocular tissue damage and related pathologies resulting from deregulation of antioxidant enzyme activity and marked GSH depletion. Besides, GSH turnover dynamics in the conjunctival epithelium have aroused great interest in recent years, given its implication in protecting and maintaining the ocular surface homeostasis in contexts like inflammatory diseases and oxidative stress conditions. In this view, our study can pave the way in exploring the use of *L.SK* as an endogen antioxidant system inducer, especially for GSH replenish in preventing and treating inflammatory, age-related, and oxidative stress-based conditions.
